# Public Attitudes Toward Notification of Use of Artificial Intelligence in Health Care

**DOI:** 10.1001/jamanetworkopen.2024.50102

**Published:** 2024-12-11

**Authors:** Jodyn Platt, Paige Nong, Gloria Carmona, Sharon Kardia

**Affiliations:** 1 Department of Learning Health Sciences, University of Michigan Medical School, Ann Arbor; 2 Division of Health Policy and Management, University of Minnesota School of Public Health, Minneapolis; 3 Department of Epidemiology, University of Michigan School of Public Health, Ann Arbor

## Abstract

This survey study examines public expectations related to notification about the use of artificial intelligence (AI) in health care.

## Introduction

Patient notification, a longstanding minimum standard in clinical and research ethics, is central to data privacy laws and informed consent. As artificial intelligence (AI) applications increase across industries, policy frameworks highlight transparency via notification as a core component of appropriate AI use.^1,2^ However, requirements and policies for such notifications are not well established, and health systems vary in their commitment to transparency.^3^ Evidence on public expectations related to notification can support policymakers and health systems in priority setting.

## Methods

From June 27 to July 17, 2023, we conducted a survey (including a video describing how AI is used in health care and scenario-based questions that provided examples) of the US public on their attitudes about AI in health care. The survey was validated using cognitive interviews and stakeholder review. We used the National Opinion Research Center (NORC) AmeriSpeak Panel, a probability-based sample of people living in the US. Black or African American and Hispanic respondents were oversampled to ensure adequate power to detect differences across groups. The NORC institutional review board (IRB) obtained written informed consent from panel participants; the survey used did not require additional informed consent from either the NORC or University of Michigan IRBs. The eMethods in Supplement 1 provides survey execution details. This study followed the AAPOR reporting guideline.

We asked participants how true it was that “It is important that I am notified about the use of AI in my health care.” Options were (1) not at all true, (2) somewhat true, (3) fairly true, and (4) very true. Responses to that question overall and by demographic group (sex, age, race and ethnicity, and education) were weighted using poststratification survey weights derived from the Current Population Survey to produce national estimates of desire for notification of AI in health care. Stata/IC, version 16, was used for analysis. *P* < .05 was considered significant.

## Results

Our survey found 4.8% (95% CI, 3.7%-6.2%) of adults (n = 99) did not find notification important. Over half (62.7%; CI, 60.0%-65.8% [n = 1267]) stated it was very true they want to be notified (Figure). The weighted mean score of desire for notification was 3.39 (95% CI, 3.33-3.44). The maximum difference in mean score was 0.13 by sex (*P* = .02), 0.43 by age (*P* < .001), 0.25 by race and ethnicity (*P* = .002), and 0.37 by education (*P* < .001) (Table).

**Figure.**
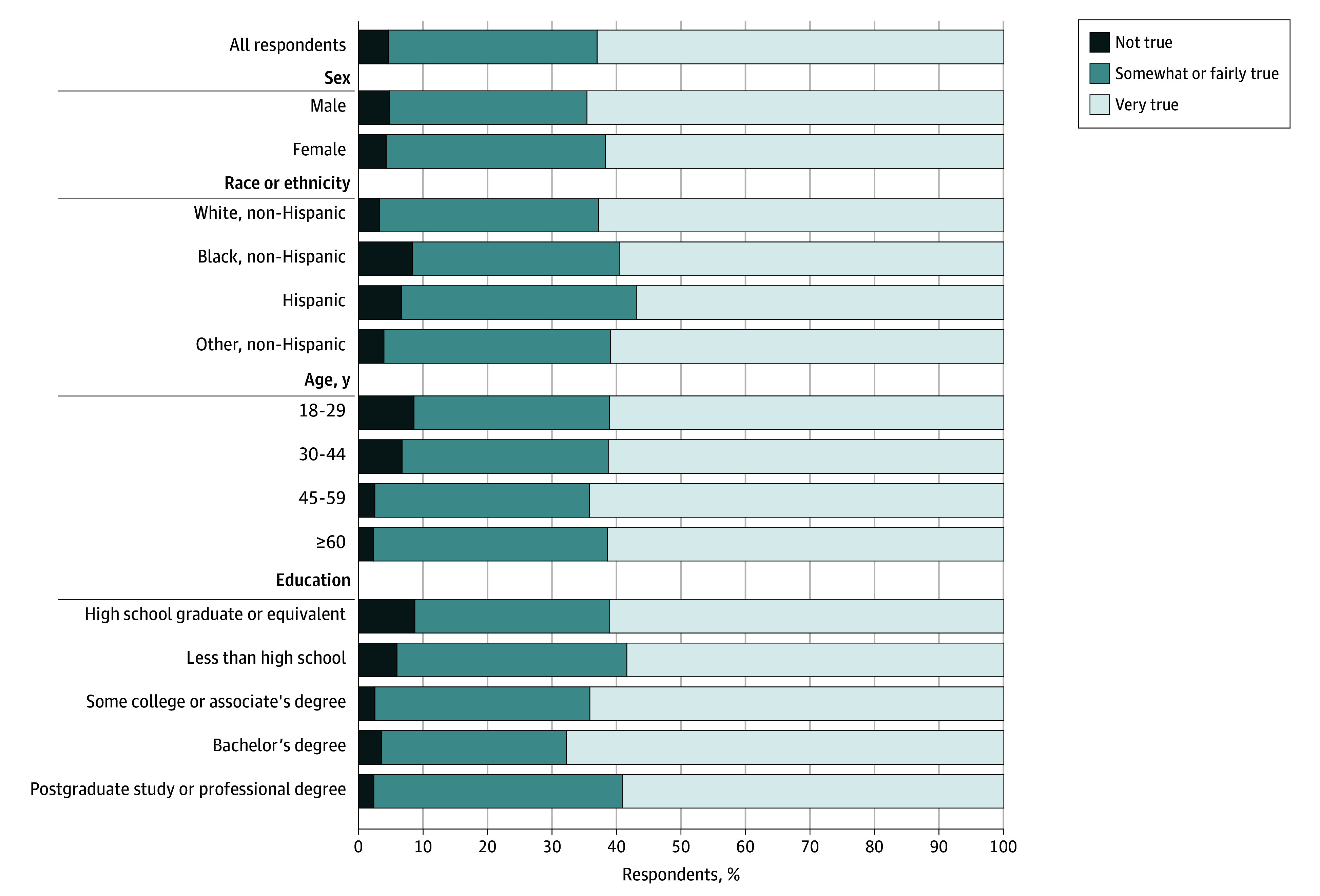
Weighted Proportion of US Population That Would Like to Be Notified About the Use of Artificial Intelligence in Their Health Care (N = 2021) Bar chart representing desire for notification among adults (aged ≥18 years) living in the US. Proportions are weighted using poststratification survey weights derived from the Current Population Survey. Weighted percentages and numbers are listed for responses overall and by demographic characteristic to the question: how true is it that “It is important that I am notified about the use of artificial intelligence in my healthcare.” Response options were (1) not at all true, (2) somewhat true, (3) fairly true, and (4) very true.

**Table.** Demographic Characteristics of Sample and Weighted Mean of Desire for Notification About the Use of Artificial Intelligence in Health Care Among Adults Living in the US

Characteristic	Unweighted frequency, No. (%)	Weighted mean of notification (95% CI)^a^	Maximum difference (*P* value)^b^
Overall (total sample)	2021 (100)	3.39 (3.33-3.44)	NA
Sex			
Male	962 (47.6)	3.32 (3.25-3.40)	0.13 (.02)
Female	1059 (52.4)	3.45 (3.37-3.53)	
Age, y			
18-29	321 (15.9)	3.14 (3.00-3.28)	0.43 (<.001)
30-44	592 (29.3)	3.28 (3.18-3.38)	
45-59	462 (22.9)	3.49 (3.38-3.59)	
≥60	646 (32.0)	3.57 (3.48-3.65)	
Race and ethnicity			
Black, non-Hispanic	533 (26.4)	3.21 (3.10-3.33)	0.25 (.002)
Hispanic	516 (25.5)	3.28 (3.19-3.37)	
White, non-Hispanic	871 (43.1)	3.46 (3.39-3.53)	
Other, non-Hispanic^c^	101 (5.0)	3.33 (3.11-3.55)	
Education			
Less than high school	141 (7.0)	3.14 (2.94-3.35)	0.37 (<.001)
High school graduate or equivalent	342 (16.9)	3.20 (3.07-3.33)	
Some college or associate’s degree	826 (40.9)	3.52 (3.45-3.59)	
Bachelor’s degree	410 (20.3)	3.50 (3.40-3.59)	
Postgraduate study or professional degree	302 (14.9)	3.51 (3.39-3.63)	

Abbreviation: NA, not applicable.

^a^ Question: how true is it that “It is important that I am notified about the use of artificial intelligence in my health care”? Scale, 1 to 4 (where 1 indicates not at all true and 4 indicates very true).

^b^ Test of null hypothesis that weighted means are equal (adjusted Wald test).

^c^ Includes people who reported race and ethnicity as Asian, non-Hispanic; 2 or more races; or did not specify.

## Discussion

A previous study examined the public’s preferences for notification about use of health information and biospecimens.^4^ That study, using the same scale, found preferences for notification about use of health information were slightly higher than for notification about use of biospecimens (mean score, 3.15 [95% CI, 3.12-3.19] vs 3.13 [95% CI, 3.10-3.17]; *P* = .01). In this study, desire for notification about AI in health care was higher than that for either health information or biospecimens.

With public opinion research, there is unmeasured error, and our study is limited as a cross-sectional analysis, calling for future longitudinal studies to examine change over time. Nonetheless, our findings suggest that notification about AI will be necessary for ethical AI and should be a priority for organizations and policymakers. With this signal about the public’s preference for notification, the question for health systems and policymakers is not whether to notify patients but when and how. As health systems begin to establish governance for AI tools, multiple approaches to notification will be needed.^5^

Our findings indicate differences in demographic groups, particularly in the ethical context of historical, structural, and systemic inequity. Female respondents expressed greater desire than male respondents for notification. White respondents expressed greater desire than Black or African American respondents for notification, suggesting that notification may be necessary but not sufficient.^6^ Collaborative efforts that engage the public, patients, and experts on the range of AI applications should support comprehensive, evidence-based programs that promote transparency about AI in health care to ensure trustworthiness of health systems.

## Notes

Supplement 1.
eMethods.

Supplement 2.
Data Sharing Statement
