# Supplementary material for: Public Attitudes Toward Notification of Use of Artificial Intelligence in Health Care
Source: JAMA Netw Open. 2024 Dec 11;7(12):e2450102. doi: 10.1001/jamanetworkopen.2024.50102 (PMC11635529; doi:10.1001/jamanetworkopen.2024.50102)
Supplement: Supplement 1. — eMethods. [file jamanetwopen-e2450102-s001.pdf]

# Supplementary Online Content

Platt J, Nong P, Carmona G, Kardia S. Public attitudes toward notification of use of artificial intelligence in health care. *JAMA Netw Open.* 2024;7(12):e2450102. doi:10.1001/jamanetworkopen.2024.50102

**eMethods**

This supplementary material has been provided by the authors to give readers additional information about their work.

© 2024 Platt J et al. *JAMA Network Open.*

**eMethods.**

NORC at the University of Chicago

# ARTIFICIAL INTELLIGENCE FOR CLINICAL DECISION SUPPORT 2023 UNIVERSITY OF MICHIGAN

AmeriSpeak

## PROJECT METHODS AND TRANSPARENCY REPORT

August 11, 2023

**Client Contact:** Jody Platt
**NORC Account Manager:**
Lauren Squires | Squires-Lauren@norc.org
**NORC Project Manager:**
David Reisner | Reisner-David@norc.org

Research You Can Trust™

© 2024 Platt J et al. *JAMA Network Open.*

# STUDY INTRODUCTION

NORC conducted the Artificial Intelligence for Clinical Decision Support (AI-CDS) on behalf of Jody Platt using NORC's AmeriSpeak® Panel for the sample source. This research was done to measure behaviors and attitudes related to the use of artificial intelligence in healthcare.

The survey was offered in English-only, and was self-administered by the respondent online via the Web.

AAPOR Transparency Initiative
AMERICAN ASSOCIATION FOR PUBLIC OPINION RESEARCH

**AAPOR Transparency Initiative**

This *AmeriSpeak Project Methods and Transparency Report* provides complete information on how the survey was executed, including any information disclosure to meet the requirement of the AAPOR Transparency Initiative. NORC at the University of Chicago is a Charter Member of the AAPOR Transparency Initiative, which fosters open science of survey research by acknowledging those organizations that pledge to practice transparency in their reporting of survey-based research findings. More on the Transparency Initiative can be found here: https://www.aapor.org/Standards-Ethics/Transparency-Initiative/FAQs.aspx

**NORC Card**

This *AmeriSpeak Project Methods and Transparency Report* combines into one document two previous deliverables: The NORC Card and The Project Report. AmeriSpeak designed the NORC Card to meet the requirements of the AAPOR Transparency Initiative. The Project Report addressed steps taken to build the AmeriSpeak Panel and conduct the study. Thus, the information in the NORC Card and the Project Report is now fully provided in this *AmeriSpeak Project Methods and Transparency Report*. We at NORC are proud of our rigorous scientific research methodology, and we are deeply committed to transparency in the work we do and the insights we deliver. If there is any information in your *AmeriSpeak Project Methods and Transparency Report* that needs further clarification, please reach out to us so we can provide you with the answers you need.

© 2024 Platt J et al. *JAMA Network Open.*

# SURVEY OVERVIEW

**Study Target Population:** General Population Age 18+
**Sample Units:** 9,548
**Completed Units:** 2,039
**Expected Eligibility Rate:** 100%
**Observed Eligibility Rate:** 100%
**Margin of Error:** ±2.97 percentage points (pp)
**Design Effect:** 1.87
**Survey Field Period:** June 27, 2023 – July 17, 2023
**Median Duration (minutes):** 22

**Definitions of the above categories:**

*Study Target Population:* The total set of individuals of interest to which the researcher intends to generalize their conclusions.

*Sample Units:* The number of panel members selected into the study sample.

*Completed Units:* The number of sample units that completed the interview based on the study-specific definition of what constitutes a complete interview. This number excludes any cases where an interviewer finished a survey, but the case was removed due to data quality concerns (the process for such removal is detailed later in this report).

*Expected Eligibility Rate:* The percentage of the sampling population who are expected to meet study eligibility criteria.

*Observed Eligibility Rate:* The percentage of the sample members who were eligible for the study among those who answered the screening questions.

*Design Effect:* The design effect is the variance under the complex design divided by the variance under an SRS (simple random sampling) design of the same sample size. The reported design effect is an approximation based on the coefficient of variation of the final survey weights.

*Margin of Error:* Margin of error is defined as half the width of the 95% confidence interval for a proportion estimate of 50% adjusted for design effect. It is therefore the largest margin of error possible for all estimated percentages based on the study sample.

*Survey Field Length:* the period from the earliest to the latest contact dates of cases sampled for the survey.

*Duration:* Length of time for completed interviews. Interview length is calculated differently depending upon whether the interview was conducted over the phone or via the web. For telephone mode, it is the time from when the respondent picks up the telephone until they hang up the telephone. For web interviews, it is the time from when they first connect to the web system to the time they log off the system or become inactive. In the case of multiple contacts, this number represents the sum of those contacts.

# STUDY-SPECIFIC DETAILS

## Sampling

An 18+ year old general population sample was selected from NORC's AmeriSpeak Panel for this study. In addition, oversamples for African Americans and Hispanics were drawn.

The sample for a specific study is selected from the AmeriSpeak Panel using sampling strata based on age, race/Hispanic ethnicity, education, and gender (48 sampling strata in total). Sample selection takes into account the expected differential survey completion rates across the sampling strata. The size of the selected sample per stratum is determined such that the distribution of the complete surveys across the strata matches that of the target population as represented by census data. If a panel household has more than one active adult panel member, only one adult panel member is selected at random. When panelists are selected for an AmeriSpeak survey, the selection process, within each sampling strata,

© 2024 Platt J et al. *JAMA Network Open.*

favors those who were not selected in the most recent previous AmeriSpeak survey. This selection process is designed to minimize the number of surveys any one panelist is exposed to and maximize the rotation of all panelists across AmeriSpeak surveys.

For more detailed information on the AmeriSpeak panel recruitment and management methodology, please see the Appendix ("Technical Overview of the AmeriSpeak® Panel NORC'S Probability-Based Household Panel") attached to this AmeriSpeak Project Report.

An oversample of 500 African American and another oversample of 500 Hispanic panelists were collected for this study.

The following outcome measures are for race/ethnicity when looking at the entire sample, including both the oversample and general population portions of the sample:

**African American:**

**Completed Sample Units (n):** 540
**Margin of Error:** ±5.38 percentage points (pp)
**Design Effect:** 1.63

**Hispanic:**

**Completed Sample Units (n):** 519
**Margin of Error:** ±6.03 percentage points (pp)
**Design Effect:** 1.96

**White/All Other:**

**Completed Sample Units (n):** 980
**Margin of Error:** ±3.83 percentage points (pp)
**Design Effect:** 1.50

## Field

A small sample of English-speaking AmeriSpeak web-mode panelists were invited on May 15 for a pretest. In total, NORC collected116 pretest interviews. The initial data from the pretest was reviewed by NORC and delivered to Jody Platt.

The survey was truncated before fielding the Main survey to collect the surveys interviews used for the final data. The pretest ran significantly over the expected completion duration requiring the need for editing. Pretest interviews are not included in the final data.

For the main survey, a sub-sample of AmeriSpeak web-mode panelists were invited to the survey on June 27 in a soft-launch. The initial data from the soft-launch was once again reviewed to confirm that there are no processing or programming errors. Once reviewed, the remainder of sampled AmeriSpeak panelists were invited to the survey on July 17.

In total, NORC collected 2,039 of final interviews. This does not include interviews that may have been removed for data quality purposes (see below).

This final collection of survey completers includes specific oversamples of Non-Hispanic African Americans (540 of completions) and Hispanics (519 of completions) to ensure adequate sample size of those groups for analysis. These oversampled groups are weighted down to match their respective proportion in the population in the weighting process (see description of that process later in this report).

© 2024 Platt J et al. *JAMA Network Open.*

## Panel & Survey Sample Performance

To meet requirements in the AAPOR Transparency Initiative, we offer performance outcome measures of both the AmeriSpeak Panel and the sample selected from the panel. The AmeriSpeak Panel is a household panel, so recruitment and retention rates are household rates. The survey sample is an individual-level sample pulled from the AmeriSpeak panel, so those are individual-level rates.

| Panel Outcome Measures | |
|---|---|
| Weighted Household Panel Recruitment Rate (WPRecr) | Weighted Household Panel Retention Rate (WPRet) |
| 20.7% | 79.9% |

*Weighted Household Recruitment Rate (WPRecr):* The weighted AAPOR RR3[1] at the household level for AmeriSpeak panel recruitment. A recruited household is a household where at least one adult successfully completed the recruitment survey and joined the panel.

*Weighted Household Retention Rate (WPRet):* The weighted percent of recruited households that remain on the panel and are available for sampling for this survey. Unavailable panelists are those who have temporarily or permanently asked to be removed from the panel or from receiving surveys.

| Survey Sample Outcome Measures | |
|---|---|
| Survey Completion Rate (SurC) | Weighted Cumulative Response Rate (WCR) |
| 21.4% | 3.5% |

*Survey Completion Rate (SurC):* The percent of sample members who completed the survey interview. 9,548 panelists were invited to the survey, and 2,039 completed the survey. As noted earlier, survey completes exclude any cases removed due to data quality concerns.

*Weighted Cumulative Response Rate (WCR):* The overall survey response rate that accounts for survey response in all phases, including panel recruitment, panel retention, and survey completion. This overall rate is weighted to account for the sample design and differential inclusion probabilities of sample members in all sampling stages. *(WCR=SurC x WPRet x WPRecr)*

## Gaining Cooperation of AmeriSpeak Panelists for the Study

If invited, AmeriSpeak panelists can take the survey online through the password-protected AmeriSpeak Mobile App, the password-protected AmeriSpeak Web portal, or by following a link in the e-mail invitation sent to them.

To encourage study cooperation, NORC sent the initial invitation and email reminders to sampled web-mode panelists on the following dates:

- June 22
- June 23

[1]AAPOR RR3 and other response rate calculations can be found here: https://www.aapor.org/Education-Resources/For-Researchers/Poll-Survey-FAQ/Response-Rates-An-Overview.aspx.

© 2024 Platt J et al. *JAMA Network Open.*

- June 26
- July 1
- July 6
- July 11
- July 12

Panelists were offered the cash equivalent of $4 for completing this survey.

## Data Processing & Data Quality Review

NORC prepared a fully labeled data file of respondent survey data and demographic data for Jody Platt.

NORC applied cleaning rules to the survey data for quality control. In total, 95 cases were removed from the final set of completed interviews based on three cleaning rules. Descriptions of the cleaning criteria and the counts from each are below (counts are overlapping).

- Removing Speeders (i.e., those that completed the survey in less than one-third the median duration)
    - 74 removed for speeding
- Removing Respondents with High Refusal Rates (i.e., those that skip or refused more than 50% of the eligible questions)
    - 50 removed for high refusal rates

Of those 95 cases removed:

- 66 cases were marked with one of the two flags above
- 29 cases were marked with both of the two flags above

AmeriSpeak is a probability-based panel, where respondents must be chosen by us to join, where access to surveys is controlled by the panelist secure log-in information to a web portal or app. E-mails, text invitations, or interview-operated telephone calls go directly to the address/number of the recruited panelist. When being called by phone, the panelist is requested by name. The way AmeriSpeak surveys are programmed and panelists are invited, panelists cannot take the survey more than once, and each panelist is always identifiable based on a unique ID. For these reasons, AmeriSpeak does not suffer the problem of "bots," fabricated profiles, non-invited respondents, or individuals or members of the household repeatedly and illegitimately taking the same survey.

## Statistical Weighting

The final weight variable that is delivered with the data is a product of three weights:

***AmeriSpeak Panel Weights:*** Weights developed for all panel members to account for their probability of selection into the sample of panel recruits, panel recruitment nonresponse adjustments, and poststratification adjustments of the recruited panel to match population benchmarks.

***Study Specific Base Weights:*** Sampling weights developed for a study sample selected from the panel to account for their selection probabilities under the sample design. The base weights are a product of the AmeriSpeak Panel Weights and the inverse of selection probabilities associated with sample selection from the panel.

***Study Specific Final Weights:*** These are final weights developed for all competed cases of a specific study. The final weights are adjustments of the base weights to address survey nonresponse through a weighting class method. Raking adjustment are then applied to the non-response adjusted weights to align the survey sample to specific population benchmarks. The final weights may be trimmed to reduce the influence of extreme weights on survey estimates.

The following information goes deeper into the specifics of each of the weights.

© 2024 Platt J et al. *JAMA Network Open.*

***AmeriSpeak Panel Weights:*** Since the sampling frame for this study is the AmeriSpeak Panel, which itself is a sample, the starting point of the weighting process for the study is the AmeriSpeak panel weight[2]. To develop the panel weight, NORC first computed the panel base weight as the inverse of the probability of selection from the NORC National Frame (the sampling frame that is used to sample housing units for AmeriSpeak) or other address-based sample frames (supplemental panel samples were selected from frames developed from the USPS Delivery Sequence Files). The sample design and recruitment protocol for the AmeriSpeak Panel involve unequal sampling rates across the sampling strata and additional subsampling of initial nonresponding housing units for in-person nonresponse follow-up (NRFU). The panel base weights reflect all the variations in panel sample selection probabilities. The panel base weights are then adjusted to account for unknown eligibility and nonresponse among eligible housing units. These adjustments were conducted using weighting classes defined by some household characteristics provided by commercial data vendors, including partisan score, political party identification, the presence of young adult(s), and minority status. To produce the final household panel weights, the household-level nonresponse adjusted weights are post-stratified to match the number of households per census division obtained from the most recent Current Population Survey (CPS). Final household weights are assigned to each eligible adult in the recruited household. These person-level weights are then adjusted to compensate for nonresponding adults within a recruited household. Finally, the nonresponse adjusted person-level panel weights are raked to population totals associated with the following variables:

**Variables & the Variable Categories for Panel Recruitment Non-Response Raking**

- Age: 18-24, 25-29, 20-39, 40-49, 50-59, 60-64, and 65+
- Gender: Male and Female
- Census Division: New England, Middle Atlantic, East North Central, West North Central, South Atlantic, East South Central, West South Central, Mountain, and Pacific
- Race/Ethnicity: Non-Hispanic White, Non-Hispanic Black, Hispanic, and Non-Hispanic Other
- Education: Less than High School, High School/GED, Some College, and BA and Above
- Housing Tenure: Home Owner and Other
- Household phone status: Cell Phone-only, Dual User, and Landline-only/Phoneless
- Age x Gender: 18-34 Male, 18-34 Female, 35-49 Male, 35-49 Female, 50-64 Male, 50-64 Female, 65+ Male, and 65+ Female
- Age x Race/Ethnicity: 18-34 Non-Hispanic White, 18-34 All Other, 35-49 Non-Hispanic White, 35-49 All Other, 50-64 Non-Hispanic White, 50-64 All Other, 65+ Non-Hispanic White, and 65+ All Other

The external population totals are obtained from the Current Population Survey, except for Household Phone Status, which is determined by the National Institutes of Health bi-annual survey on wireless substitutions.[3] The weights adjusted to the external population totals are the *final panel weights*.

***Study Specific Base Weights:*** These are developed to adjust for unequal selection probabilities from the AmeriSpeak panel, differential nonresponse across subpopulations, and frame coverage limitations. All these weighting adjustments are applied to the final panel weights described above.

The sample for this study is selected from the AmeriSpeak Panel using sampling strata (see the description of the sampling strata for this study earlier in this report). Sample selection takes into account the expected differential survey completion rates across these strata based on average completion rates in previous surveys. This sample selection based on expected nonresponse ensures a more representative final sample of completed interviews. However, the net result of the sampling

---

[2] The AmeriSpeak panel weight existed prior to this study; the weighting procedures are described here for clarity and completeness.

[3] Blumberg SJ, Luke JV. Wireless substitution: Early Release of Estimates from the National Health Interview Survey, January-June 2021. National Center for Health Statistics. November 2021. Available from: https://www.cdc.gov/nchs/nhis.htm

© 2024 Platt J et al. *JAMA Network Open.*

design is an unequal selection probability that varies depending on the strata a respondent represents. *Study-specific base weights* are computed as the product of the final panel weights and the inverse of the probabilities of selection under the study sample design.

Finally, ***Study Specific Final Weights*** are created by first adjusting the base weights for survey nonresponse through a weighting class method, where the weighting classes are defined by age, race/ethnicity, gender, and education. After that, a raking ratio adjustment is applied to the nonresponse adjusted base weights to align the sample with known population benchmarks made up of the topline socio-demographic characteristics of the following:

**Variables & the Variable Categories for Study-Specific Survey Non-Response Raking**

Age: Non-Hispanic White/All Other 18-24, Non-Hispanic White/All Other 25-29, Non-Hispanic White/All Other 30-39, Non-Hispanic White/All Other 40-49, Non-Hispanic White/All Other 50-59, Non-Hispanic White/All Other 60-64, Non-Hispanic White/All Other 65+, Non-Hispanic Black 18-24, Non-Hispanic Black 25-29, Non-Hispanic Black 30-39, Non-Hispanic Black 40-49, Non-Hispanic Black 50-59, Non-Hispanic Black 60-64, Non-Hispanic Black 65+, Hispanic 18-24, Hispanic 25-29, Hispanic 30-39, Hispanic 40-49, Hispanic 50-59, Hispanic 60-64, and Hispanic 65+

Gender: Non-Hispanic/All Other White Male, Non-Hispanic/All Other White Female, Non-Hispanic Black Male, Non-Hispanic Black Female, Hispanic Male, and Hispanic Female

Region: NH White/All Other Northeast, NH White/All Other Midwest, NH White/All Other South, NH White/All Other West, NH Black Northeast, NH Black Midwest, NH Black South, NH Black West, Hispanic Northeast, Hispanic Midwest, Hispanic South, and Hispanic West

Education: Non-Hispanic/All Other Less than High School, Non-Hispanic/All Other High School/GED, Non-Hispanic/All Other Some College, Non-Hispanic/All Other BA and Above, Non-Hispanic Black Less than High School, Non-Hispanic Black High School/GED, Non-Hispanic Black Some College, Non-Hispanic Black BA and Above, Hispanic Less than High School, Hispanic High School/GED, Hispanic Some College, and Hispanic BA and Above

Age x Gender: NH White/All Other 18 - 34 Male, NH White/All Other 18 - 34 Female, NH White/All Other 35 - 49 Male, NH White/All Other 35 - 49 Female, NH White/All Other 50 - 64 Male, NH White/All Other 50 - 64 Female, NH White/All Other 65+ Male, NH White/All Other 65+ Female, NH Black 18 - 34 Male, NH Black 18 - 34 Female, NH Black 35 - 49 Male, NH Black 35 - 49 Female, NH Black 50 - 64 Male, NH Black 50 - 64 Female, NH Black 65+ Male, NH Black 65+ Female, Hispanic 18 - 34 Male, Hispanic 18 - 34 Female, Hispanic 35 - 49 Male, Hispanic 35 - 49 Female, Hispanic 50 - 64 Male, Hispanic 50 - 64 Female, Hispanic 65+ Male, Hispanic 65+ Female

These sociodemographic characteristics are weighted to benchmarks from the 2023 February Current Population Survey.

Raking and re-raking are done during the weighting process such that the weighted demographic distribution of the survey completes resemble the demographic distribution in the target population. The assumption is that the key survey items are related to the demographics. Therefore, by aligning the survey respondent demographics with the target population, the key survey items should also be in closer alignment with the target population.

At the final stage of the weighting process, any extreme weights are trimmed based on a criterion of minimizing the mean squared error associated with key survey estimates. Weights after trimming are re-raked to the same population totals to produce the ***final study weights***.

### Additional Oversample Weights

This survey includes an oversample of non-Hispanic African Americans and Hispanics which were weighted down to its proportions in the overall population in the final main study weights. Some survey

© 2024 Platt J et al. *JAMA Network Open.*

packages are not able to recognize weight variations and do not leverage the full potential of an oversample when testing for statistical significance. The basic SPSS package (without the additional Complex Samples Module) has this limitation, while SAS, Stata, and most R packages do not. Since we are delivering this data in an SPSS format, we have included a second weight variable to address this. The oversample variable in the delivered data has the following variable name: WEIGHT2. The weight values in an oversample weight variable scale up the oversampled group(s) to their actual unweighted sample size. Analyzing the data using this weight variable should only occur when analyzing the oversampled group or any subgroup that is wholly composed of the oversampled group, or when comparing the oversampled with a group outside of that oversample. It is inappropriate to use the oversample weight variable when analyzing the overall survey sample or any subgroup that overlaps (does not fit completely within or without) an oversampled group. Using this weight variable in this inappropriate way will lead to incorrect results that are skewed toward the results of the oversampled groups. It is important to note that, when analyzing the oversampled group, results will be the same whether one is using the oversample weight variable or the main weight variable. This difference is limited to the margin of error attained in data from the oversample and non-oversampled groups. Without the use of this weight, the margin of error for the oversampled group would be (typically) much larger than the true value, and the margin of error for the non-oversampled group would be lower. In addition, as the main weight will reduce the effective sample size of the oversampled group, it can be the case that using this weight would lead to significant rounding errors, particularly in oversampled of very small populations (e.g., 5%).

© 2024 Platt J et al. *JAMA Network Open.*

## Benchmark Comparisons

The following table shows the weighted and unweighted estimates for key demographics and compares them to population benchmarks.[4]

| Demographic Category | Subcategory | Unweighted (%) | Weighted (%) | Benchmark (%) |
|---|---|---|---|---|
| Age | 18 - 34 | 27.7 | 29.0 | 29.0 |
| | 35 - 49 | 25.1 | 24.4 | 24.4 |
| | 50 - 64 | 23.9 | 24.2 | 24.2 |
| | 65 Plus | 23.4 | 22.4 | 22.4 |
| Race/Ethnicity | Non-Hispanic White | 43.0 | 63.1 | 61.4 |
| | Non-Hispanic Black | 26.5 | 12.1 | 12.1 |
| | Hispanic | 25.5 | 17.4 | 17.4 |
| | Non-Hispanic Asian/Pacific Islander | 2.6 | 5.0 | 6.8 |
| | Non-Hispanic Others | 2.5 | 2.4 | 2.2 |
| Education Status | Less than High School | 7.0 | 9.0 | 9.0 |
| | High School Equivalent | 16.8 | 29.0 | 29.0 |
| | Some College/Associate Degree | 41.0 | 26.4 | 26.4 |
| | Bachelor's Degree | 20.3 | 20.6 | 22.5 |
| | Graduate Degree | 15.0 | 15.0 | 13.1 |
| Sex | Male | 47.3 | 48.8 | 48.8 |
| | Female | 52.7 | 51.2 | 51.2 |
| Age by Race | NH White/All Other 18 - 24 | 3.0 | 7.3 | 7.3 |
| | NH White/All Other 25 - 29 | 2.9 | 5.4 | 5.4 |
| | NH White/All Other 30 - 39 | 8.0 | 11.5 | 11.5 |
| | NH White/All Other 40 - 49 | 7.8 | 10.6 | 10.6 |
| | NH White/All Other 50 - 59 | 7.2 | 11.4 | 11.4 |
| | NH White/All Other 60 - 64 | 4.7 | 6.3 | 6.3 |
| | NH White/All Other 65 + | 14.4 | 18.2 | 18.2 |
| | NH Black 18 - 24 | 2.3 | 1.6 | 1.6 |
| | NH Black 25 - 29 | 1.9 | 1.2 | 1.2 |
| | NH Black 30 - 39 | 6.0 | 2.3 | 2.3 |
| | NH Black 40 - 49 | 4.3 | 2.0 | 2.0 |
| | NH Black 50 - 59 | 4.5 | 1.9 | 1.9 |
| | NH Black 60 - 64 | 2.6 | 1.0 | 1.0 |
| | NH Black 65 + | 5.0 | 2.1 | 2.1 |
| | Hispanic 18 - 24 | 2.7 | 2.8 | 2.8 |
| | Hispanic 25 - 29 | 3.0 | 1.9 | 1.9 |
| | Hispanic 30 - 39 | 6.0 | 3.6 | 3.6 |

[4] Because we trim the weights to remove extreme weights and hold down weight variation, the final study weights may end up deviating from exact populations benchmarks by small but acceptable amounts. Even without trimming, there can be a limit in the ability to perfectly match benchmarks along all variables and categories included in the raking procedure. Our goal is to rake as close as possible before trimming.

© 2024 Platt J et al. *JAMA Network Open.*

| | | | | |
|---|---|---|---|---|
| | Hispanic 40 - 49 | 4.6 | 3.3 | 3.3 |
| | Hispanic 50 - 59 | 3.8 | 2.7 | 2.7 |
| | Hispanic 60 - 64 | 1.2 | 1.0 | 1.0 |
| | Hispanic 65 + | 4.1 | 2.1 | 2.1 |
| **Education by Race** | NH White/All Other < HS | 2.4 | 3.9 | 3.9 |
| | NH White/All Other HS | 6.7 | 18.9 | 18.9 |
| | NH White/All Other > HS < college | 17.9 | 18.7 | 18.7 |
| | NH White/All Other >= college | 21.1 | 28.9 | 28.9 |
| | NH Black < HS | 2.4 | 1.1 | 1.1 |
| | NH Black HS | 5.1 | 4.1 | 4.1 |
| | NH Black > HS < college | 11.0 | 3.6 | 3.6 |
| | NH Black >= college | 8.0 | 3.3 | 3.3 |
| | Hispanic < HS | 2.3 | 4.0 | 4.0 |
| | Hispanic HS | 5.0 | 5.9 | 5.9 |
| | Hispanic > HS < college | 12.0 | 4.1 | 4.1 |
| | Hispanic >= college | 6.2 | 3.4 | 3.4 |
| **Sex by Race** | NH White/All Other Male | 23.4 | 34.5 | 34.5 |
| | NH White/All Other Female | 24.6 | 36.0 | 36.0 |
| | NH Black Male | 11.6 | 5.6 | 5.6 |
| | NH Black Female | 14.9 | 6.5 | 6.5 |
| | Hispanic Male | 12.3 | 8.8 | 8.8 |
| | Hispanic Female | 13.2 | 8.7 | 8.7 |
| **Region by Race** | NH White/All Other Northeast | 8.2 | 13.1 | 13.1 |
| | NH White/All Other Midwest | 14.6 | 17.0 | 17.0 |
| | NH White/All Other South | 14.0 | 24.3 | 24.3 |
| | NH White/All Other West | 11.3 | 16.0 | 16.0 |
| | NH Black Northeast | 3.0 | 1.9 | 1.9 |
| | NH Black Midwest | 7.4 | 2.0 | 2.0 |
| | NH Black South | 13.8 | 7.1 | 7.1 |
| | NH Black West | 2.3 | 1.1 | 1.1 |
| | Hispanic Northeast | 2.3 | 2.4 | 2.4 |
| | Hispanic Midwest | 4.4 | 1.5 | 1.5 |
| | Hispanic South | 9.2 | 7.0 | 7.0 |
| | Hispanic West | 9.6 | 6.6 | 6.6 |
| **Age x Gender by Race** | NH White/All Other 18 - 34 Male | 5.9 | 9.3 | 9.3 |
| | NH White/All Other 18 - 34 Female | 5.1 | 9.1 | 9.1 |
| | NH White/All Other 35 - 49 Male | 5.3 | 8.1 | 8.1 |
| | NH White/All Other 35 - 49 Female | 5.6 | 8.1 | 8.1 |
| | NH White/All Other 50 - 64 Male | 5.5 | 8.7 | 8.7 |
| | NH White/All Other 50 - 64 Female | 6.4 | 8.9 | 8.9 |
| | NH White/All Other 65 + Male | 6.8 | 8.3 | 8.3 |

© 2024 Platt J et al. *JAMA Network Open.*

| | | | | |
|---|---|---|---|---|
| | NH White/All Other 65 + Female | 7.6 | 9.8 | 9.8 |
| | NH Black 18 - 34 Male | 3.4 | 1.9 | 1.9 |
| | NH Black 18 - 34 Female | 4.1 | 2.1 | 2.1 |
| | NH Black 35 - 49 Male | 3.3 | 1.4 | 1.4 |
| | NH Black 35 - 49 Female | 3.7 | 1.6 | 1.6 |
| | NH Black 50 - 64 Male | 2.6 | 1.3 | 1.3 |
| | NH Black 50 - 64 Female | 4.4 | 1.5 | 1.5 |
| | NH Black 65 + Male | 2.3 | 0.9 | 0.9 |
| | NH Black 65 + Female | 2.7 | 1.3 | 1.3 |
| | Hispanic 18 - 34 Male | 4.5 | 3.4 | 3.4 |
| | Hispanic 18 - 34 Female | 4.8 | 3.2 | 3.2 |
| | Hispanic 35 - 49 Male | 3.5 | 2.6 | 2.6 |
| | Hispanic 35 - 49 Female | 3.6 | 2.5 | 2.5 |
| | Hispanic 50 - 64 Male | 2.6 | 1.8 | 1.8 |
| | Hispanic 50 - 64 Female | 2.4 | 1.8 | 1.8 |
| | Hispanic 65 + Male | 1.6 | 0.9 | 0.9 |
| | Hispanic 65 + Female | 2.5 | 1.2 | 1.2 |

As a part of the AAPOR Transparency Initiative, it is incumbent on us to state that there are no perfect studies, and all research and methods have their limitations. The purpose of this document is to make apparent, for this study, some possible limitations, the steps taken to minimize them, and the potential or known sources of measurable or estimated error whenever possible. However, there is always going to be some unmeasured and unknowable error with all forms of public opinion research, including ours.

## Deliverables

The following files were created for Jody Platt as part of the study deliverables:

- Survey interview data file in both SPSS and STATA formats
- Codebook in an Excel format
- Final questionnaire – in a complete programming format, in Word document
- Final questionnaire – in a simpler format (standard AmeriSpeak intro and outro language, programming language, Spanish (if relevant), and CATI version or interviewer instruction (if relevant) are removed), in Word document
- Project report documenting study procedures and information on the AmeriSpeak Panel

© 2024 Platt J et al. *JAMA Network Open.*

# How to Describe AmeriSpeak and NORC @ the University of Chicago

For purposes of publication, when describing the AmeriSpeak Panel and its methodology, we recommend using the following language:

> Funded and operated by NORC at the University of Chicago, **AmeriSpeak®** is a probability-based panel designed to be representative of the US household population. Randomly selected US households are sampled using area probability and address-based sampling, with a known, non-zero probability of selection from the NORC National Sample Frame. These sampled households are then contacted by US mail, telephone, and field interviewers (face to face). The panel provides sample coverage of approximately 97% of the U.S. household population. Those excluded from the sample include people with P.O. Box only addresses, some addresses not listed in the USPS Delivery Sequence File, and some newly constructed dwellings. While most AmeriSpeak households participate in surveys by web, non-internet households can participate in AmeriSpeak surveys by telephone. Households without conventional internet access but having web access via smartphones are allowed to participate in AmeriSpeak surveys by web. AmeriSpeak panelists participate in NORC studies or studies conducted by NORC on behalf of governmental agencies, academic researchers, and media and commercial organizations.
>
> For more information, email AmeriSpeak-BD@norc.org or visit AmeriSpeak.norc.org.

If editors or reviewers are requesting anything more specific or any other detail, please reach out to us to make certain you are using accurate language.

For a less technical, panel-specific description of **AmeriSpeak**, we recommend:

**AmeriSpeak** is the first U.S. multi-client household panel to combine the speed and cost-effectiveness of panel surveys with enhanced representativeness of the U.S. population, an industry-leading response rate, and an innovative and thorough Project Methods and Transparency Report. Since its founding by NORC at the University of Chicago in 2015, AmeriSpeak has produced more than 1000 surveys, been cited by dozens of media outlets, and become the primary survey partner of the nation's preeminent news service, The Associated Press. AmeriSpeak is the most scientifically rigorous multi-client panel available in the U.S. market. Amerispeak.norc.org.

**NORC at the University of Chicago** is best described as follows:

NORC at the University of Chicago conducts research and analysis that decision-makers trust. As a nonpartisan research organization and a pioneer in measuring and understanding the world, NORC has studied almost every aspect of the human experience and every major news event for more than eight decades. Today, NORC partners with government, corporate, and nonprofit clients around the world to provide the objectivity and expertise necessary to inform the critical decisions facing society. www.norc.org

Please refer to the full name "NORC at the University of Chicago" when first mentioning us. Using simply "NORC," thereafter, is fine. Our name is now only the acronym and does not need to be spelled out.

© 2024 Platt J et al. *JAMA Network Open.*

**APPENDIX**

# TECHNICAL OVERVIEW OF THE AMERISPEAK® PANEL NORC'S PROBABILITY-BASED HOUSEHOLD PANEL

Updated February 8, 2022

This technical overview provides the basic information about AmeriSpeak®, a large probability-based panel funded and operated by NORC at the University of Chicago. AmeriSpeak is designed to be representative of the U.S. household population, including all 50 states and the District of Columbia. U.S. households are randomly selected with a known, non-zero probability from the NORC National Frame as well as address-based sample (ABS) frames, and then recruited by mail, telephone, and by field interviewers face to face. AmeriSpeak panelists participate in NORC studies or studies conducted by NORC on behalf of governmental agencies, academic institutions, the media, and commercial organizations.

The construction of the AmeriSpeak panel started in 2014 with pilot samples. In 2015, about 7,000 households were recruited from a sample of around 60,000 addresses. In 2016, about 128,000 addresses were sampled to expand the panel to around 20,000 recruited households. About 51,000 addresses were selected for the 2017 recruitment, which led to the expansion of the regular AmeriSpeak panel to 23,000 recruited households. The AmeriSpeak Panel expanded to approximately 30,000 households in 2018 and 35,000 households in 2019 through further recruitment efforts. The current panel size is 54,001 panel members aged 13 and over residing in over 43,000 households.

In addition to the regular panel for general population studies, AmeriSpeak also contains sub-panels to support studies of special populations, including AmeriSpeak Latino, AmeriSpeak Teen, and AmeriSpeak Young Adult 18-34 (which features an oversample of African Americans, Hispanics, and Asians). AmeriSpeak is also the probability sample source for TrueNorth®, the NORC calibration solution for combining probability and non-probability samples for estimation through small area modeling that leverages data from AmeriSpeak, the American Community Survey, Current Population Survey, and other data sources for improved statistical efficiency.[5] AmeriSpeak is also the sample source for the Foresight 50+ panel, which is a partnership between AARP and NORC that provides a high-quality panel for organizations looking for insights from older adults living in the United States.[6]

## Panel Sample Frame

The primary sampling frame for AmeriSpeak is the 2010 NORC National Frame, a multistage probability sample that fully represents the U.S. household population. We provide a brief description of how the National Frame was constructed after the 2010 Census.

The primary sampling units (PSUs) in the first stage sample selection are 1,917 National Frame Areas (NFAs), each of which is an entire metropolitan area (made up of one or more counties), a county, or a group of counties with a minimum population of 10,000. A total of 126 NFAs are selected in the first stage, including 38 certainty NFAs, 60 urban NFAs, and 28 non-urban NFAs. The largest 38 NFAs, those with a population of at least 1,543,728 (0.5 percent of the 2010 Census U.S. population), were selected into the National Frame with certainty.

Within the 126 selected NFAs, the secondary sampling units (SSUs) are segments defined from Census tracts or block groups, where each segment contains at least 300 housing units according to the 2010 Census. Within the certainty NFAs, a sample of 896 segments was selected using systematic PPS sampling, where the size of a segment is the number of housing units. Implicit stratification was achieved

[5] For more information about TrueNorth, see http://amerispeak.norc.org/our-capabilities/Pages/TrueNorth.aspx.
[6] For more information about Foresight 50+, see https://www.norc.org/Research/Capabilities/Pages/Foresight50.aspx

© 2024 Platt J et al. *JAMA Network Open.*

by sorting the segments by location (NFA, state, and county), principal city indicator, and by ethnic and income indicators. From each urban and rural NFA, a sample of 8 and 5 segments was selected, respectively, using systematic PPS sampling where the measure of size is the number of housing units per segment. A total of 618 segments are selected from the non-certainty NFAs[7]. Overall, a stratified probability sample of 1,514 segments was selected into the National Frame in the second stage sampling.

Within the selected segments, all housing units are listed using the U.S. Postal Service Delivery Sequence File (DSF). In the 123 segments where the DSF coverage is deemed inadequate, the DSF address list is enhanced with an in-person field listing to improve coverage. The final National Frame, consisting of all listed households in the sample segments, is estimated to provide over 97 percent coverage of the U.S. household population. It contains almost 3 million households, including over 80,000 rural households that are added through the in-person listing. In addition to NORC's National Frame, the DSF is used as a supplemental sample frame in four states. Although nationally representative, the National Frame does not include households from Alaska, Iowa, North Dakota, and Wyoming. Since 2016, the annual panel recruitment sample has included a small address-based sample from these four states to assure AmeriSpeak presence in all U.S. States and Washington, D.C.

In 2017, an enhanced DSF frame was also used to develop a new Latino Panel with adequate representation of Spanish-language-dominant Hispanics. Census tracts with a high incidence (at least 30%) of Spanish-dominant Hispanics were targeted for this recruitment. Furthermore, within these Census tracts, households that were flagged as Hispanic based on consumer vendor data (that are typically used for direct-mail marketing) were oversampled.

### Panel Sample Selection

For panel sample selection between 2014 and 2018 and in 2020, National Frame segments were stratified into six sampling strata based on the race/ethnicity and age composition of each segment, as below:

- Hispanic, high youth segments
- Hispanic, not high youth segments
- Non-Hispanic Black, high youth segments
- Non-Hispanic Black, not high youth segments
- Other, high youth segments
- Other, not high youth segments

Hispanic segments are those where Hispanics make up at least a third of the population and the Hispanic share in the population is greater than that of non-Hispanic Black. Similarly, non-Hispanic Black segments are those where non-Hispanic Black make up at least a third of the population and the non-Hispanic Black share in the population is greater than that of Hispanics. Finally, High Youth refers to segments in which 18-24-year-old adults are at least 12% of the total adult population. The above stratification is used to oversample housing units in areas with a higher concentration of young adults, Hispanics, and non-Hispanic African Americans. The resulting household sample is referred to as the initial AmeriSpeak sample or sample for initial panel recruitment.

To support the second stage of panel recruitment, initially sampled but nonresponding housing units are subsampled for a nonresponse follow-up (NRFU)[8]. At this stage, consumer vendor data are matched to

[7] A sample of 5 segments was selected from each of the 28 non-urban NFAs. However, 2 sample segments were later subsampled out in Montana due to cost.
[8] A small fraction of initially nonresponding housing units is not eligible for NRFU, including "hard refusals" and those with an appointment for a call back from NORC.

© 2024 Platt J et al. *JAMA Network Open.*

the pending housing units, and housing units that are flagged as having a young adult[9] (18-34 years of age) or minority (Hispanic[10], non-Hispanic Black[11]) are oversampled for the NRFU sample. Overall, approximately one in five initially nonresponding housing units are subsampled for NRFU using the same six sampling strata defined above. Due to NRFU, these initially nonresponding housing units have a higher selection probability compared to the housing units that were recruited during the first stage of panel recruitment.

A two-phase state-based ABS sample design was used for the 2019 AmeriSpeak recruitment. NORC's National Frame is designed to represent the U.S. household population nationally. At the state level, however, the panel may have more significant clustering effects from the use of the National Frame, especially for states with a small population. The primary objective of the 2019 design is to improve state-level representation by selecting the recruitment sample mostly from areas that are outside the National Frame. A stratified systematic sample was selected in the first phase, where each state constitutes a sampling stratum, and the sample was allocated to the strata proportional to the square root of the state population. In the second phase, young adults, Hispanic, non-Hispanic Black, and conservatives are oversampled based on commercial data sources to improve their representation in the panel. Because the 2019 design did not use NRFU face-to-face recruitment, the 2019 design did not involve geographic clustering.

In 2020 we returned to the "standard" sampling strategy employed in 2014 through 2018, with intentions to conduct a robust NRFU. However, the COVID-19 pandemic prevented NORC from utilizing field interviewers and the NRFU was limited to its usual first stage, a Federal Express mailing to 20% of the total sample. After an analysis of state-level representativity after 2019 recruitment, it was determined that further statewide representativity was needed in four states: WI, MO, WA, and CO. As such, statewide samples using the USPS DSF file were generated for supplemental recruitment.

In 2021, NORC also recruited into an AmeriSpeak probability sample of persons aged 50 and older using a random national consumer address file (estimated 96% sample coverage of all households in the U.S.). AmeriSpeak re-empaneled approximately 6,000 study participants in this initiative.

It was clear at the start of 2021 that NORC would not immediately be able to conduct in-person interviewing given the ongoing COVID-19 pandemic. However, NORC sought to test new sampling strategies (noted below) early in 2021 in the hopes of documenting their efficacy and continuing and improving on them for the rest of 2021. Additionally, it was hoped that NORC would be able to conduct in-person interviewing in the second half of 2021. As such, the 2021 recruiting sample was split into five replicates, the first of which utilized DSF sample, and was released early in the calendar year, while future replicates were sampled using the NORC National Frame and were held until mid-year for recruiting.

At the end of 2020, a major assessment of panel representativeness was conducted to inform the 2021 sampling strategy. This analysis again explored representativity by state, but as well explored a full range of demographic variables. This analysis was conducted both with the full panelist dataset as well as by assessing "effective panelists," a measure of the likely demographic distributions that would occur among complete cases in any typical AmeriSpeak survey. This analysis found that AmeriSpeak could benefit from additional panelists in seven groups: households earning over $200,000, household with children, Hispanics, Hispanics that specifically speak Spanish, African Americans, persons ages 18 to 24, and persons with less than a High School education. As such, the sample was stratified using NORC Big Data Classifiers (Dutwin et al, 2022), a technique utilizing available consumer and other public Big Data to

[9] A young adult flagged household refers to a household where MSG or TargetSmart indicated there was an 18-24-year-old adult in the household. In 2016 and 2017, a slightly different definition was used, and a young adult flagged household was defined as having an 18–34-year-old adult in the household by MSG or 18–30-year-old adult by TargetSmart.

[10] A Hispanic flagged household refers to a household where MSG or TargetSmart indicated the presence of a Hispanic adult in the household.

[11] A non-Hispanic Black-flagged household refers to a household where MSG or TargetSmart indicated the presence of a non-Hispanic Black adult in the household.

© 2024 Platt J et al. *JAMA Network Open.*

make predictions on a range of household attributes during survey sampling. Households predicted to be one of these seven attributes were oversampled, while households predicted only hold persons aged 50 and older, or otherwise not predicted hold someone with one of the seven attributes, were under sampled. This sampling method was tested in the first sampling replicate, and given very positive results, was continued in all other 2021 replicates.

NORC's strategy of "waiting it out" was effective, as the sample replicates released mid-year allowed NORC, to wait for an effective "COVID window" to conduct in-person interviewing. In short, in-person interviewing commenced after the peak of the Delta variant in 2021 and concluded with the peak of the Omicron variants. NORC was able to conduct a full NRFU in-person effort during this time.

### Panel Recruitment Procedures

AmeriSpeak Panel recruitment is a two-stage process: (i) initial recruitment using USPS mailings, telephone contact, and modest incentives, and (ii) a more elaborate NRFU recruitment using FedEx mailings, enhanced incentives, and in-person visits by NORC field interviewers.

For the initial recruitment, sample households are invited to join AmeriSpeak online by visiting the panel website AmeriSpeak.org or by calling a toll-free telephone line (inbound/outbound supported). Both English and Spanish languages are supported for online and telephone recruitment. The initial recruitment data collection protocol features the following: an over-sized pre-notification postcard, a USPS recruitment package in a 9"x12" envelope (containing a cover letter, a summary of the privacy policy, FAQs, and a study brochure), two follow-up postcards, and contact by NORC's telephone research center for sample units with a matched telephone number.

For the second stage NRFU recruitment, a stratified random sample is selected from the nonrespondents of the initial recruitment. Units sampled for NRFU are sent a new recruitment package by Federal Express with an enhanced incentive offer. Shortly thereafter, NORC field interviewers make personal, face-to-face visits to the pending cases to encourage participation. Once the households are located, the field interviewers administer the recruitment survey in-person using CAPI or else encourage the respondents to register online or by telephone.

### Panel Recruitment Response Rate and Other Panel Statistics

A sample household is considered recruited or responded if at least one adult in the household joins the panel. The weighted household response rate (AAPOR RR3) is about 6% for initial recruitment and 28% for NRFU recruitment. We report two recruitment response rates: one for all the panel recruitment years (2014-2021) and one for the recruitment years with NRFU (2014-2018 and 2021). For all recruitment years, the cumulative weighted household response rate is 21.9%; for recruitment years with NRFU, and the cumulative weighted household response rate is 34.0%.[12] For client studies requiring a panel recruitment response rate exceeding 30%, the sampling frame may be restricted to the panelists recruited in the NRFU years. The panel recruitment response rate calculation methodology is consistent with AAPOR guidelines and fully documented.[13] The annual panel retention rate is about 85%.

For individual client surveys based on the AmeriSpeak Panel, the AAPOR RR3 response rate is between 10% to 20% depending on specific study parameters such as target population, survey length, time in the field, salience of subject, and the like. This response rate takes into account panel recruitment rate, panel retention rate, and survey participation rate.[14]

---

[12] As the 2021 NRFU is continuing to wind down, response rates noted here are estimated for 2021 sample cases.
[13] See http://amerispeak.norc.org/research/Pages/WhitePaper_ResponseRateCalculation_AmeriSpeak_2016.pdf
[14] A properly calculated cumulative AAPOR response rate for panel-based research takes into account all sources of non-response at each stage of the panel recruitment, management, and survey administration process (see https://www.aapor.org/AAPOR_Main/media/publications/Standard-Definitions20169theditionfinal.pdf, page 48-9). A common misapplication of the term "response rate" in online panel surveys is to represent the survey-specific cooperation rate as the

© 2024 Platt J et al. *JAMA Network Open.*

Other important panel statistics with respect to the 2014-2019 and 2021 recruited households are as follows: 68% are recruited in the initial stage and 32% are recruited via NRFU; 92% of the active panelists prefer to do web or online surveys, while 8% prefer to participate in telephone surveys; 16% of the recruited households are non-Internet[15]; 82% are cell phone only or cell phone mostly; 17% are African-American and 18% Hispanic; and 29% have household income below $30,000 (compared to CPS benchmark of 26%).[16]

### Impact of Non-Response Follow-Up

NRFU is instrumental in producing the industry-leading response rate for AmeriSpeak Panel recruitment. Moreover, due to the more intensive effort, NRFU recruitments better represent hard-to-reach groups and are therefore more representative of the target population. For example, initial recruitment tends to under-represent young adults 18-34 years of age. NRFU recruitment corrects for this bias by bringing the age distribution of the panel closer to ACS benchmarks.

Overall, NRFU recruitment significantly improves the representation of the panel with respect to demographic segments that are under-represented among the respondents to the initial recruitment, including young adults (persons 18 to 34 years of age), African Americans, Hispanics, lower-income households, renters, cellphone-only households, and persons with lower educational attainment (e.g., no college degree). To the extent that these demographic characteristics are correlated with substantive survey variables, NRFU helps to reduce potential non-response bias in the sample estimates. NORC's research indicates that NRFU respondents are indeed somewhat different from initial respondents for many common survey variables. For example, compared to the panelists recruited during the initial stage, NRFU panelists tend to be more conservative politically, more likely to attend church, less interested in current events or topics in the news report, less knowledgeable about science, less likely to be in favor of gun control policies, less likely to read a print newspaper (more likely to read the news online and use social media), more likely to eat at fast-food restaurants, and so on[17]. These observations illustrate that NRFU recruitment is critical for achieving a more balanced panel and for making the substantive estimates in AmeriSpeak studies more accurate. Even though NRFU panelists are more reluctant to complete surveys, the addition of NRFU panelists reduced total absolute bias on average 5 to 21 percentage points when compared to the initial stage recruits (among examined surveys).[18]

### Mixed-Mode Data Collection

The AmeriSpeak Panel supports mixed-mode data collection to improve response rate and the representativeness of the complete surveys. During the recruitment survey, AmeriSpeak panelists are offered an opportunity to choose their preferred mode—web or phone—for future participation in AmeriSpeak surveys. A recruited household can consist of both web- and phone-mode panelists. Panelists predominantly prefer web over phone mode. As of February 2020, 92% of the active panelists prefer to do web or online surveys, while 8% prefer to participate in telephone surveys. The telephone mode encompasses panelists without internet access, panelists whose only internet access is via a smartphone, and panelists with internet access but are unwilling to share an email address.

---

"cumulative survey response rate." *See* "Response Rate Calculation Methodology for Recruitment of a Two-Phase Probability-Based Panel: The Case of AmeriSpeak" authored by Robert Montgomery, J. Michael Dennis, N. Ganesh. The paper is available at https://amerispeak.norc.org/research/.

[15] The non-internet households (HHs) are those that do not select "High-speed, broadband internet at home (such as cable or DSL)" or "Dial-up internet at home" response options when they are asked "What kind of internet access do you have? Please select all that apply" item in the recruitment survey. The non-internet HHs include those that only use internet on a cell connection or mobile phone.

[16] For transparency purposes, unweighted percentages are presented in this section. Hence, these results do not take into account selection probabilities. The base weighted distributions that take into account selection probabilities can be provided upon request.

[17] See "The Undercounted: Measuring the Impact of 'Nonresponse Follow-up' on Research Data and Outcome Measures" authored by Ipek Bilgen, J. Michael Dennis, N. Ganesh. The paper will be soon available at https://amerispeak.norc.org/research/.

[18] *See* "Nonresponse Follow-up Impact on AmeriSpeak Panel Sample Composition and Representativeness" authored by Ipek Bilgen, J. Michael Dennis, N. Ganesh. The paper is available at https://amerispeak.norc.org/research/.

© 2024 Platt J et al. *JAMA Network Open.*

To the extent that non-internet households or "net averse" persons are different from the rest of the population, mixed-mode surveys have better population coverage and produce more accurate population estimates. NORC's telephone interviewers administer the telephone surveys using a data collection system supporting both the phone and web modes, providing an integrated sample management and data collection platform. For panelists using smartphones for web-mode surveys, the NORC survey system renders an optimized presentation of the survey questions for these mobile users.

### Panel Management and Maintenance

Panel management and maintenance are crucial for panel health and efficiency. NORC maintains strict panel management rules to limit respondent burden, reduce panel attrition, and minimize the risk of panel fatigue. On average, AmeriSpeak panelists are invited to participate in client studies two to three times a month. AmeriSpeak works with NORC clients to create surveys that provide an appropriate user experience for AmeriSpeak panelists. AmeriSpeak will not field surveys that in our professional judgment will result in a poor user experience for our panelists. AmeriSpeak also has a designated website and a telephone number for panelist communications.

Panel maintenance is a dynamic process because the AmeriSpeak Panel is supplemented and refreshed regularly over time to grow the panel, compensate for panel attrition, and improve panel representation for specific subpopulations. For example, the Latino Panel and Teen Panel are created to support studies of Hispanics and teenagers, respectively; the 2019 recruitment is primarily designed to improve sample representation at the state level. As panelists are added or/and removed from the panel, the panel refreshment process takes place to ensure that the refreshed panel fully represents the corresponding target population.

## ADDITIONAL RESOURCES

To learn more about AmeriSpeak or to share an RFP, please contact AmeriSpeak at AmeriSpeak-BD@norc.org. Information about AmeriSpeak capabilities and research papers are available online at AmeriSpeak.NORC.org.

© 2024 Platt J et al. *JAMA Network Open.*
