# Supplementary material for: Public Attitudes Toward Notification of Use of Artificial Intelligence in Health Care
Source: JAMA Netw Open. 2024 Dec 11;7(12):e2450102. doi: 10.1001/jamanetworkopen.2024.50102 (PMC11635529; doi:10.1001/jamanetworkopen.2024.50102)
Supplement: Supplement 2. — Data Sharing Statement [file jamanetwopen-e2450102-s002.pdf]

Data Sharing Statement

Platt. Public Attitudes Toward Notification of Use of Artificial Intelligence in Health Care. *JAMA Netw Open*. Published December 11, 2024. doi:10.1001/jamanetworkopen.2024.50102

Data
Data available: No

Additional Information
Explanation for why data not available: Data will be made available upon reasonable request
